# GliomaDB: A Web Server for Integrating Glioma Omics Data and Interactive Analysis

**DOI:** 10.1016/j.gpb.2018.03.008

**Published:** 2019-12-05

**Authors:** Yadong Yang, Yang Sui, Bingbing Xie, Hongzhu Qu, Xiangdong Fang

**Affiliations:** 1 CAS Key Laboratory of Genome Sciences and Information, Beijing Institute of Genomics, Chinese Academy of Sciences, Beijing 100101, China; 2 University of Chinese Academy of Sciences, Beijing 100049, China

**Keywords:** Database, Variations, Methylation, Network, Survival analysis

## Abstract

Gliomas are one of the most common types of brain cancers. Numerous efforts have been devoted to studying the mechanisms of glioma genesis and identifying biomarkers for diagnosis and treatment. To help further investigations, we present a comprehensive **database** named GliomaDB. GliomaDB includes 21,086 samples from 4303 patients and integrates genomic, transcriptomic, epigenomic, clinical, and gene-drug association data regarding glioblastoma multiforme (GBM) and low-grade glioma (LGG) from The Cancer Genome Atlas (TCGA), Gene Expression Omnibus (GEO), the Chinese Glioma Genome Atlas (CGGA), the Memorial Sloan Kettering Cancer Center Integrated Mutation Profiling of Actionable Cancer Targets (MSK-IMPACT), the US Food and Drug Administration (FDA), and PharmGKB. GliomaDB offers a user-friendly interface for two main types of functionalities. The first comprises queries of (i) somatic mutations, (ii) gene expression, (iii) microRNA (miRNA) expression, and (iv) DNA **methylation**. In addition, queries can be executed at the gene, region, and base level. Second, GliomaDB allows users to perform **survival analysis**, coexpression **network** visualization, multi-omics data visualization, and targeted drug recommendations based on personalized **variations**. GliomaDB bridges the gap between glioma genomics big data and the delivery of integrated information for end users, thus enabling both researchers and clinicians to effectively use publicly available data and empowering the progression of precision medicine in glioma. GliomaDB is freely accessible at http://bigd.big.ac.cn/gliomaDB.

## Introduction

Gliomas are the most common form of brain cancers and can be classified as Grade I–IV based on standards set by the World Health Organization (WHO). Grade I, II, and III gliomas are usually considered low-grade glioma (LGG), whereas Grade IV tumors are frequently termed high-grade glioma, which is also known as glioblastoma multiforme (GBM) (https://cancergenome.nih.gov/cancersselected/lowergradeglioma). In the United States of America, there were 23,820 estimated new cases and 17,760 estimated new deaths owing to diseases of the brain and nervous system in 2019 [1]. Glioma is one of the deadliest forms of human cancers, with a 5-year relative survival of 33% [2], and for GBM patients in particular, the median duration of survival is estimated to be 14 months after maximal surgical resection, radiotherapy, and chemotherapy [3].

Recent years have witnessed the rapid development of high-throughput technology, including microarray and next-generation sequencing. For example, The Cancer Genome Atlas (TCGA) [4], [5], [6] has been assembled from thousands of glioma cancer and noncancer samples. In addition, an enormous amount of data from independent studies has been deposited into Gene Expression Omnibus (GEO) [7], [8]; both of these data aggregates provide an unprecedented opportunity for glioma research. For example, genomic profiling could be used to separate primary and secondary GBM, which are otherwise indistinguishable histologically [9], [10]. Single cell sequencing technologies have been utilized for identifying tumor initiating cells in glioma and presenting a paradigm for interpretation of intra-tumor heterogeneity and personalized therapy [11]. *ATRX* has been associated with increased telomere length based on whole-genome data analysis; glioma molecular classification by *IDH* mutation status and 1p/19q codeletion were identified using clinically relevant molecular subsets [6]. Despite advances in glioma research, most studies use only a limited number of datasets because of insufficient ready-to-use resources. Moreover, the highly dispersed nature of data resources hindered the progression of precision medicine. Hence, an integrated database must be urgently established for the storage, retrieval, and analysis of big data in glioma.

In the recent past, several databases have been developed for the storage and analysis of big data in cancer. Some of the databases focus on pan-cancer expression analysis; for example, Gene Expression Profiling Interactive Analysis (GEPIA, http://gepia.cancer-pku.cn) [12] provides RNA sequencing (RNA-seq) data from 9736 tumors and 8587 normal samples from the TCGA and the Genotype-Tissue Expression (GTEx) projects and offers tools for differential analysis, similar gene analysis, correlation analysis, and dimensionality reduction. Cancer RNA-seq Nexus (http://syslab4.nchu.edu.tw/) [13] provides 28 types of cancer RNA-seq data from the TCGA and GEO. Moreover, this database provides functionalities for the differential analysis of genes and long noncoding RNAs (lncRNAs) as well as mRNA-lncRNA coexpression network analysis. Other databases specifically focus on glioma; some examples are given as follows. (1) The diffuse low-grade glioma (DLGG) database (http://db-gliomas-gradeii.net/) [14] provides 210 different fluid-attenuated inversion recovery (FLAIR) magnetic resonance (MR) images of DLGG patients at different levels of evolution and the tools for the analysis of clinical images. (2) GLIOMASdb (http://cgga.org.cn:9091/gliomasdb/) [15] provides RNA-seq data of 325 gliomas at different stages with different subtypes and identified progression-associated genes. (3) Xena (http://xena.ucsc.edu/) [16] provides numerous useful visualization and analysis tools for deposited omics data and secure analysis and visualization of private functional genomics data. (4) cBioPortal (http://www.cbioportal.org/) [17] provides simultaneous visualization of multiple types of genomic data from multiple data sources.

Although these databases or web tools provide abundant resources for the glioma scientific and clinical community, many additional features or functions that are often required by biologists and clinicians are not appropriately addressed by these tools and databases. For example, most of the dispersive datasets in GEO are not included in GEPIA, Cancer RNA-seq Nexus, DLGG, GLIOMASdb, Xena, or cBioPortal, which limits the data usage. While Cancer RNA-seq Nexus supports the query of a specific gene or lncRNA for the coexpression network, this database contains only the connections of the query gene/lncRNA but not the connections between all nodes. Moreover, while cBioPortal provides mutation annotation from OncoKB [18], CIViC [19], and My Cancer Genome [20], there lacks information on the mutation status for healthy populations and lacks customizable annotation of mutations for targeted drug recommendations. In addition, although GEPIA provides survival analysis based on gene expression profiles, the analysis is based on a single variable; an option to consider two or more variables (genes) is not available. Furthermore, none of these databases provide miRNA expression or DNA methylation profiles. Hence, to mitigate the aforementioned problems, we developed GliomaDB, an integrative database for glioma-related data, to complement the existing databases and web tools.

## Implementation

**Figure 1:**
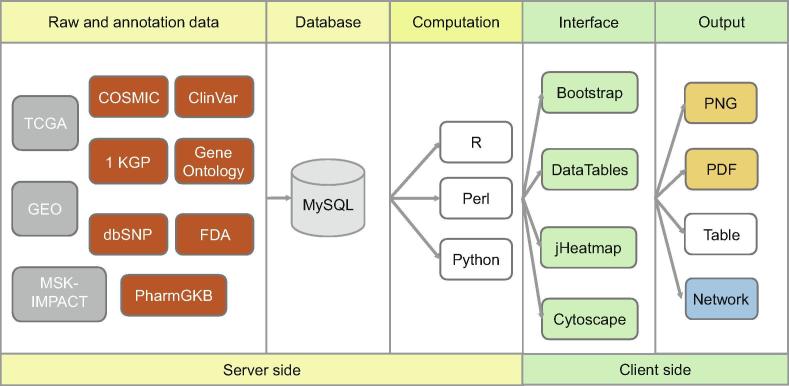
**Scheme describing data processing, storage, and display for the GliomaDB visualization tool** Raw and annotation data from 10 public databases were stored in GliomaDB and then computed or analyzed using our in-house scripts, with outputs visualized in figures or tables. TCGA, The Cancer Genome Atlas; GEO, Gene Expression Omnibus; MSK-IMPACT, Memorial Sloan Kettering-Integrated Mutation Profiling of Actionable Cancer Targets; 1 KGP, 1000 Genomes Project; COSMIC, Catalogue of Somatic Mutations in Cancer; FDA, Food and Drug Administration.

GliomaDB codes were developed using an integrated development environment, Eclipse (http://www.eclipse.org). MySQL (https://www.mysql.com) is used to store and manage the metadata information of this database. For database connection and operation, MyBatis (http://www.mybatis.org) is used as a persistence framework. Spring (http://www.springsource.org) is used for the inversion of control containers. Java Server Pages is used to render the dynamic front pages. Struts (http://struts.apache.org) is used to manage the model-view-controller model web application. GliomaDB is hosted on a CentOS operating system with two servers, with Tomcat (http://tomcat.apache.org/) serving static and dynamic content and a MySQL (version 5.6.19) server providing the features for database management. All the plotting features in GliomaDB are implemented using R (version 3.4.2), Perl (version v5.10.1), and Python (version 2.7.12). The tables for this database are generated using DataTables (https://www.datatables.net) JavaScript library. The interactive heatmap and network are visualized with jHeatmap (https://jheatmap.github.io/jheatmap/) and Cytoscape (http://js.cytoscape.org/) JavaScript library, respectively (Figure 1).

## Database content and usage

### Database structure and organization

GliomaDB comprises four modules: search, analysis, team introduction, and statistics. The search module includes four aspects: genomic mutation, gene expression, miRNA expression, and DNA methylation. The analysis module contains four analytical perspectives: survival analysis, coexpression network visualization, cluster analysis, and variant-based targeted drug recommendation.

### Data sources

Genomic variants, gene expression, miRNA expression, DNA methylation and clinical data of glioma patients were integrated from the TCGA (https://cancergenome.nih.gov/), MSK-IMPACT Clinical Sequencing Cohort [21], GEO (https://www.ncbi.nlm.nih.gov/geo/), and CGGA (http://www.cgga.org.cn/) projects; these data include tumor/normal tissue and blood samples. For the glioma sample, we selected only data from brain tissue, excluding data from cell lines, and all published data should be from after 2005. To continuously update the data, we developed a tool based on Entrez Programming Utilities (E-Utils) provided by the National Center for Biotechnology Information (NCBI) to automatically search for the newly updated datasets (see “Update” section in the “tutorial” page). For the genomic variants, we also integrated annotations from public resources, such as gene ontology (GO), the Catalogue Of Somatic Mutations In Cancer (COSMIC, http://cancer.sanger.ac.uk/cosmic/), the mutation frequency in different populations in the 1000 Genomes Project (https://www.genome.gov/27528684/1000-genomes-project/), and context information on the mutation in ClinVar (https://www.ncbi.nlm.nih.gov/clinvar/). Drug-responsive gene/variant data were collected from the Food and Drug Administration (FDA) (http://www.fda.gov/) and PharmGKB (https://www.pharmgkb.org).

### Data preprocessing

For the expression profile generated with the microarray platform from the GEO database, we first convert the probe id to the gene symbol and then use the average value to represent the expression of a gene if there are more than one probes mapped to one gene. For the data from TCGA, we first download level 3 files from the Genomic Data Commons (GDC) data portal and then link the omics data to sample and patient information with the Application Program Interface (API) (https://docs.gdc.cancer.gov/API/Users_Guide/Getting_Started/#tools-for-communicating-with-the-gdc-api) provided by the GDC data portal.

### Data statistics

GliomaDB integrates multi-omics data from 21 projects, which include 4303 patients and 21,086 samples. There are 6,083,427 records of single nucleotide variants (SNVs), and the corresponding annotations are based on hg19. There are 56,180,100 and 1,072,792 records in the gene expression and miRNA expression data, respectively. The DNA methylation data contain 27 K and 450 K data. The former has 27,578 CpG sites, and the latter has 485,578 CpG sites. There are 184,091,259 records in the DNA methylation data (Table 1). The variant/gene-related drug information collected from the FDA and PharmGKB contains data on the variant, PubMed ID, drug, disease, gene, *P* value, race, association, FDA guideline, etc. There are 77 targeted drugs and 6569 records regarding drug information.

**Table 1:** **Statistics of omics data deposited in GliomaDB**

**Data category**	**No. of projects**	**No. of samples**	**No. of patients**	**No. of records**
Somatic mutation	3	2490	1423	6,083,427
Gene expression	18	3309	3283	56,181,100
miRNA expression	3	733	715	1,072,792
DNA methylation	3	986	960	184,091,259

### Search

**Figure 2:**
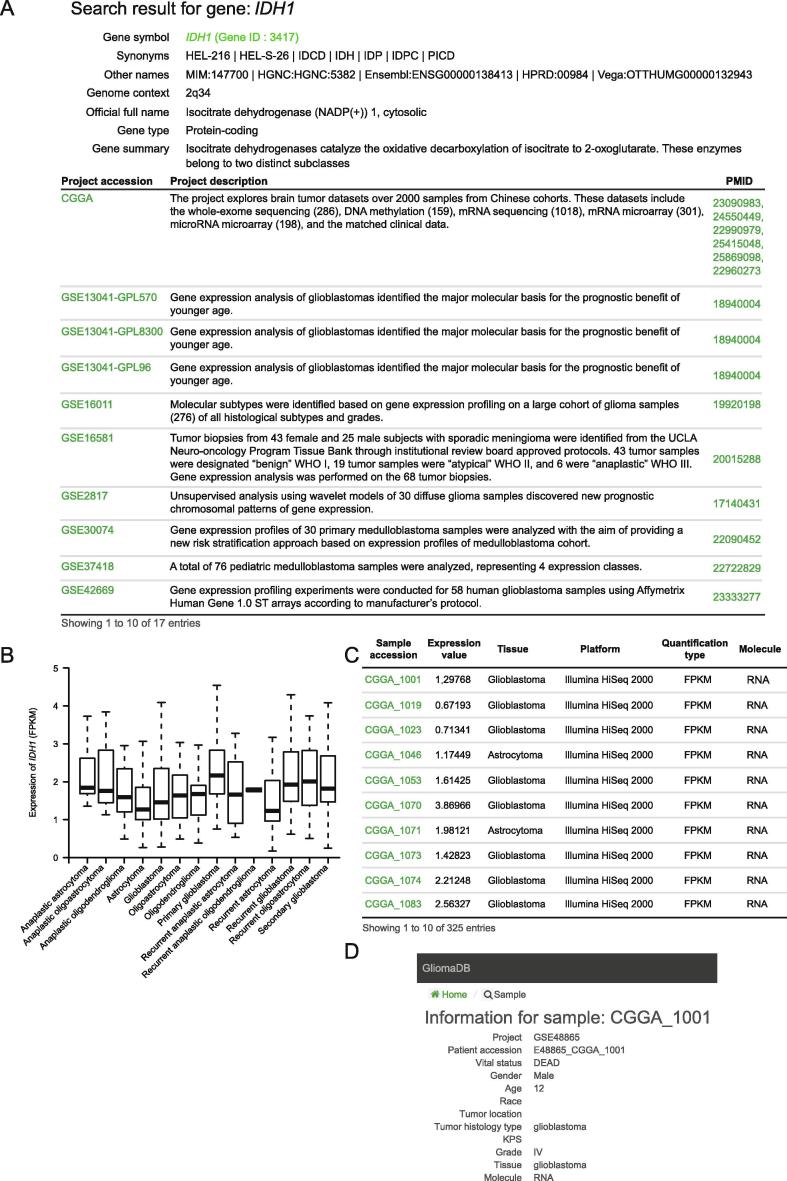
**Example of gene expression search output with *IDH1*** **A**. The first query result of the gene expression of *IDH1* is shown as an example, including the gene summary information and projects with expression records of the query gene. **B.** Boxplot showing the expression of *IDH1* in different subtype of glioma from the CGGA project. **C.** Detailed gene expression information of *IDH1* in the CGGA project (only first 10 samples are listed). **D**. Information provided for a specific sample CGGA_1001 included in the project shown in panel C. CGGA, Chinese Glioma Genome Atlas.

Four types of data can be queried in the search section: somatic mutation, gene expression, miRNA expression, and DNA methylation. GliomaDB provides a straightforward search interface. Users can query by gene symbol (*e.g.*, *IDH1*), Ensembl ID (*e.g.*, ENSG00000138413), or gene ID (*e.g.*, 3417) in the “Gene name” search field. For the mutation query, chromosomal region, gene name, and dbSNP accession number are supported for the retrieval of somatic mutations. The results include the tumor sample and the matched normal sample, which could be further linked to detailed information about the corresponding patient. We also integrated the annotation for mutations from Oncotator [22]. This mutation information included the gene information and GO categories of the gene where the mutation is located, the somatic mutations from COSMIC located in the gene, the mutation frequency of the mutation in 1000 Genomes data, and the mutation information from ClinVar. In the gene and miRNA expression search section, the results are grouped by project, considering the incompatibility of expression value between different platforms (Figure 2). The results are presented in two steps. The first step shows the summary information of the query gene and projects containing the query gene in any of its samples (Figure 2A), and the second step shows the expression boxplot of different sample groups (Figure 2B) and detailed expression (Figure 2C) of the query gene in a specific project. Users can also obtain detailed information on the patient from whom the sample originated (Figure 2D) by clicking the sample accession number in the expression search results. In the methylation search section, chromosomal region, gene name, and cgid (Infinium MethylationEPIC probe ID) are supported for the retrieval of DNA methylation, and 450 K/27 K methylation data are included.

### Analysis

**Figure 3:**
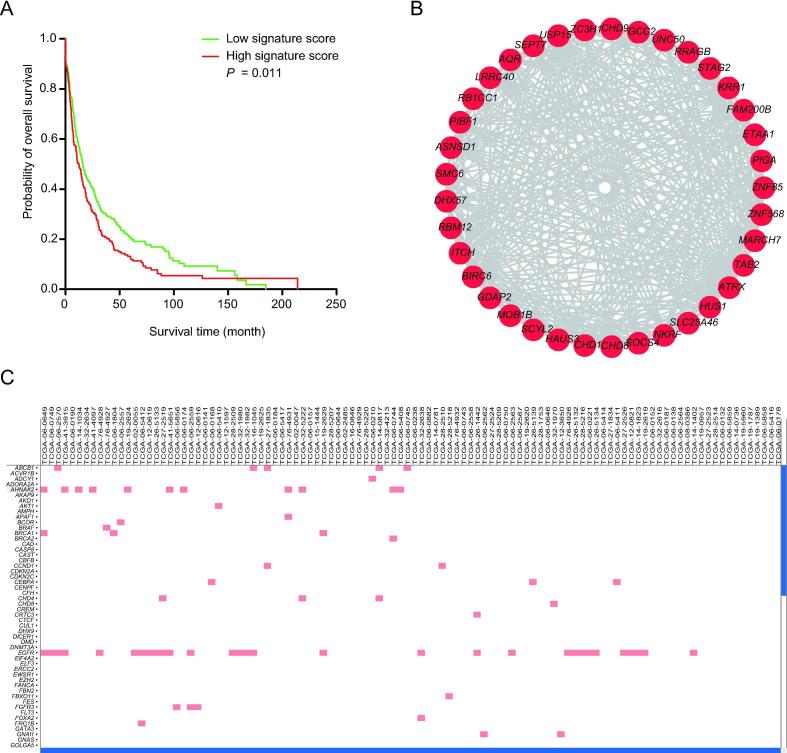
**Example of GliomaDB analysis output** **A**. The overall survival analysis of a gene/genes of interest can be calculated and presented in a Kaplan–Meier plot. Here, we use expression of two genes, *KLF1* and *NF1*, in TCGA-LGG as an example. The mean score provided by coxph is used to split the samples. Samples with scores greater than the mean score of all samples are labeled as “high signature score”, and the others are labeled as “low signature score”. *P* = 0.011 indicates that the expression of *KLF1* and *NF1* is significantly associated with the overall survival of LGG patients in TCGA-LGG. **B**. The coexpression network of genes correlated with *ATRX*. All genes (shown in red solid circles) correlated with *ATRX* were firstly selected as a dataset and the relationships of each pair of genes in the dataset are visualized with the Cytoscape plugin in different layouts. **C**. The interactive heatmap visualization of the multi-omics data tested, including mutation, copy number variation, and expression profiles.

GliomaDB includes four types of analysis: survival analysis, coexpression network, interactive heatmap visualization, and auxiliary targeted drug recommendation (Figure 3).

#### Survival analysis

Survival analysis based on gene expression levels is also widely used for predicting the clinical outcome of a given gene [23]. Therefore, the gene expression datasets were used for survival analysis. Single-gene or multiple-gene queries are both supported, and the results are presented in a Kaplan–Meier plot of two groups stratified by the mean score obtained by Cox regression (Figure 3A).

#### Coexpression network visualization

In each dataset, the Pearson’s correlation coefficient is calculated for each of the two genes, which potentially denotes their regulation relationship. For the query of one gene, the resulting network includes edges between the query gene and each of other genes with a Pearson’s correlation coefficient greater than 0.85 (Figure 3B).

#### Interactive heatmap view of multiple omics data

We integrated jHeatmap [24], an interactive web heatmap viewer built using JavaScript, to represent mutation, copy number variation, and expression profiles (Figure 3C).

#### Auxiliary targeted drug recommendation

We integrated the pharmacogenomics knowledge for personalized medicine from the FDA and PharmGKB [25] and offered a built-in interactive service for the retrieval of targeted drugs with either gene name/dbSNP accession ID/drug or a standard VCF file.

## Conclusion and discussion

GliomaDB is a web server that has been developed for the integration of multiple omics data and interactive analysis in glioma studies. The data in GliomaDB are from TCGA, the GEO database, the MSK-IMPACT project, the FDA, and PharmGKB, with thousands of tumor and normal samples included. Data types include genome, transcriptome, miRNome, methylome, targeted drug, and genetic variation-drug association. GliomaDB is a time-saving, free, and intuitive tool for tapping the full potential of publicly available genomics big data, which enables biologists and clinicians without any programming experience to obtain ready-to-use multi-omics data and perform a diverse range of data analyses. GliomaDB is designed to complement existing tools, such as cBioPortal and GEPIA. It also has the potential to become a one-stop service for data query and analysis for the scientific and clinical community associated with the glioma field. In the future, we will not only continuously update multi-omics data from both glioma and normal samples, but also develop new analytical features for further exploration of the available big genomic data. We hope that GliomaDB would facilitate a better translation of data into knowledge and of knowledge to application.

## Availability

GliomaDB is freely accessible at http://bigd.big.ac.cn/gliomaDB.

## Authors’ contributions

XF and HQ conceived the study and supervised the project. YY designed the system architecture. YY, YS, and BX wrote the source code. YY drafted the manuscript. XF revised the manuscript. All authors read and approved the final manuscript.

## Competing interests

The authors have declared no competing interests.
